# Development of a Low-Cost Optical Sensor to Detect Eutrophication in Irrigation Reservoirs

**DOI:** 10.3390/s21227637

**Published:** 2021-11-17

**Authors:** Javier Rocher, Lorena Parra, Jose M. Jimenez, Jaime Lloret, Daniel A. Basterrechea

**Affiliations:** 1 Instituto de Investigación para la Gestión Integrada de Zonas Costeras, Universitat Politècnica de València, Grao de Gandía, 46730 Valencia, Spain; jarocmo@doctor.upv.es (J.R.); loparbo@doctor.upv.es (L.P.); jojiher@dcom.upv.es (J.M.J.); dabasche@epsg.upv.es (D.A.B.); 2 Finca “El Encin”, Instituto Madrileño de Investigación y Desarrollo Rural, Agrario y Alimentario (IMIDRA), A-2, Km 38, 2, 28805 Alcalá de Henares, Spain

**Keywords:** turbidity, sediment, alga, light absorption, water quality, irrigation channel

## Abstract

In irrigation ponds, the excess of nutrients can cause eutrophication, a massive growth of microscopic algae. It might cause different problems in the irrigation infrastructure and should be monitored. In this paper, we present a low-cost sensor based on optical absorption in order to determine the concentration of algae in irrigation ponds. The sensor is composed of 5 LEDs with different wavelengths and light-dependent resistances as photoreceptors. Data are gathered for the calibration of the prototype, including two turbidity sources, sediment and algae, including pure samples and mixed samples. Samples were measured at a different concentration from 15 mg/L to 4000 mg/L. Multiple regression models and artificial neural networks, with a training and validation phase, are compared as two alternative methods to classify the tested samples. Our results indicate that using multiple regression models, it is possible to estimate the concentration of alga with an average absolute error of 32.0 mg/L and an average relative error of 11.0%. On the other hand, it is possible to classify up to 100% of the samples in the validation phase with the artificial neural network. Thus, a novel prototype capable of distinguishing turbidity sources and two classification methodologies, which can be adapted to different node features, are proposed for the operation of the developed prototype.

## 1. Introduction

Eutrophication is a process that entails the overgrown of algae and other aquatic plants due to the enrichment of a water body with nutrients and minerals. When these organisms die, they cause a reduction in dissolved oxygen in the water column. This process is a sort of pollutions that induces a loss of biodiversity in the water [1,2]. The leading causes of eutrophication are overuse of fertilizers, nutrients from animal waste, soil loss in crops, and human sewage [1]. Every water body can be eutrophicated; however, the legislation establishes areas sensible to eutrophication. In Europe, the directive 91/271/EEC defines the eutrophication sensitive areas as (I) freshwater bodies and coastal water that are eutrophic or will be in the near future if protective actions are not taken; (II) and (III) are areas where a tertiary treatment is necessary for the wastewater or another equivalent treatment. In addition, the legislation foresees the existence of less sensitive areas. These areas are water bodies with a good water flow or areas where eutrophication is unlikely [3].

The irrigation ponds used in agriculture are susceptible to eutrophication. The eutrophication of these ponds can be problematic for the ecosystems and human health. Different studies show that these ponds have an important effect on biodiversity, such as providing refuges for endangered species that live in freshwater bodies [4,5,6]. Biodiversity has essential advantages in human and agricultural environments. Biodiversity increases the environmental production of the ecosystems, increases resistance to extreme climate events [7], decreases pests [8], and reduces the transmission of infectious diseases in plants, humans, or animals [9]. The decrease in pests allows reducing the use of pesticides and minimizing the pesticide toxicology of soil that negatively affects some species that live in the soil, such as the earthworms [10]. As the overuse of fertilizers might cause eutrophication, monitoring the eutrophication in water bodies can be used to check the adequacy of farming practices of the region. The over-fertilization of the crops causes problems such as groundwater pollution and eutrophication of surrounding water bodies due to the transport of the soil by the rain [11].

Another problem linked to eutrophic environments is the synthesis and release of toxic substances. The Harmful Algal Blooms (HABs) are uncontrolled toxic algae growths affecting animals, plants, and other microscopic organisms [12]. Olson et al. [13] studied the presence of microcystin toxins in aerosol particles. They simulated the creation of aerosol with Michigan Mona Lake water, which has high levels of HAB (>200 µg/L). HAB exposure in freshwater was responsible for 34 reports of human and animal HAB-associated health events caused by 38 water bodies in Kansas [14]. On the one hand, the presence of HAB waters can be dangerous for farmers working near wells. On the other hand, the death of animals produces health problems due to their decomposition in the field and odor problems.

The monitorization of water bodies using sensors have been widely studied, and it is the best option for the continuous control of water quality or for preventing further ecological damages. In [15], the use of a sensor based on optical elements to control the turbidity in water canalizations is described. Their result has proven that the use of the optical sensor is suitable for turbidity monitoring. Another optical sensor application in water monitoring is presented in [16], where authors demonstrate that optical sensors can be employed for oil monitoring. In [17], optical fiber bragg gratings are used to monitor the mixture of oils with water. Due to the difference in density between water and oil. Controlling the turbidity is an appropriate low-cost alternative for evaluating the status of the eutrophication process. Nonetheless, the traditional turbidity measure by itself is not enough to estimate eutrophication. It is necessary to establish the source of the turbidity, which can be sediment of algae, in which case we might have eutrophication. In [18], a turbidity sensor capable of distinguishing the sediment and the algae concentration was presented. Although with this sensor, we can affirm if sediments or algae cause the turbidity, generally turbidity is caused by both sources in irrigation ponds. Therefore, it is vital to analyze if it is possible to identify the amount of turbidity due to sediment and algae to define if the water is under the eutrophication process. As far as we connect, no one paper has presented a sensor capable of analyzing this process considering the two different turbidity sources.

In this paper, we present a low-cost sensor for monitoring the presence of algae and sediments. Our prototype is based on LEDs of different colors (blue, green, yellow, orange, and red). The light from the LEDs traverses a glass containing the water we want monitoring and impacts a light-dependent resistor (LDR). The sensor uses a voltage divider to convert the values of resistance of LDR into a voltage. This voltage is read by a microprocessor and transformed into the concentration of sediment or algae to estimate eutrophication. Our prototype uses an Arduino for powering the LEDs, processes the change in the values of the LDR, and sends an alarm (if needed). Eight concentrations of sediment, algae, and combinations of both sources are analyzed to convert the voltage into the water quality parameter. The gathered values of the output voltage are evaluated using statistical software to define the accuracy of the obtained mathematical model. Our sensor can be used for monitoring the presence of eutrophication in water.

The rest of the paper is structured as follows. In Section 2, the related work is outlined. Then, the proposed system is described in Section 3. Following, Section 4 presents the methodology and materials that we use in prototype construction and data gathering. Section 5 presents and discusses the obtained results of the experiment. Finally, the conclusion and future work are summarized in Section 6.

## 2. Related Work

In this section, we present different methods for monitoring the presence of eutrophication in water bodies. There are different approaches to measuring eutrophication, from complex approaches that include measuring several chemical variables to more straightforward methods based on remote sensing.

Lin et al. [19] proposed the use of a Technique for Order Preference by Similarity to an Ideal Solution (TOPSIS) method and Monte Carlo Simulation (MCS) for evaluating the eutrophication level in lakes. TOPSIS is a multicriteria decision method to select an alternative. They used the concentration of chlorophyll-a, total nitrogen, Secchi disc, total phosphorus, and the chemical oxygen demand as criteria. They divided the values of eutrophication in 5 level (>20, >40, >60, >80, >100). The MCS is used for reducing the uncertainty of the data collection. They used this methodology in Lake Erhai and concluded that the results are consistent with the values of eutrophication. Their results pointed out that chemical oxygen demand and Secchi disc are the most sensitive factors. In another work, Yao et al. [20] used a multidimensional similarity cloud model (MSCM) to determine a lake’s eutrophication. In this paper, the criteria are the same as the one described in [19]. In this model, the authors use a randomized weighting method to process the information to reduce the effect of possible errors in the values obtained of monitoring criteria. The data are introduced in a reverse cloud generation to obtain cloud digital characteristics, and results are compared with the different eutrophic levels. The problem with these methods [19,20] is that they need some data about the chemical composition of the water. These analyses require the use of reagents with a high cost, and in situ analyses should be performed precluding the remote monitoring. The system we propose simplifies monitoring since measures are taken without requiring reagents, and the sensor can be left in the water and send the data.

The use of remote sensing is an alternative to monitoring eutrophication in lakes. One of the satellites used in remote sensing is the use of Landsat satellite. Zhou et al. [21] used the images of Landsat to measure the chlorophyll in Lake Taihu (China). They demonstrated that the TM4 band is the one with the better single-band relationship. Nevertheless, the TM1, TM2, and TM4/TM3 ratio bands have the best correlation (R^2^ = 0.809). With this method, they can monitor concentrations between 5 to 100 mg chlorophyll a/m^3^. This model has been adapted by Sòria-Perpinyà et al. [22] to monitor Valencia’s Albufera (Spain). They used the image of Landsat 5 and 7. In this case, TM1 and TM2 are not used, and they have a Pearson correlation coefficient of 0.83 and an R^2^ of 0.93. Another algorithm was proposed by Ha et al. [23]. They used the Moderate Resolution Imaging Spectroradiometer (MODIS) in the ratio of green and blue band reflectance (bands 9 and 12). The images obtained of MODIS were pre-processed to reduce the specular reflections at the sea surface and atmosphere. In addition, they used an ordinary kriging method to improve the pixel resolution to 100 m. They used a histogram minimum method (dark-object subtraction). The paper’s objective is the measurement of Chl-a in a shallow lake (Tien Yen Bay in northern Vietnam). In these cases, they measure the presence of chlorophyll in the water surfaces. Xue et al. [24] proposed a method to estimate the vertical distribution of Chl-a. They used MODIS (terra and aqua). They calculated the Normalized Difference algal Bloom Index (NDBI) (green and red bands) combined with wind speed value. These values are inserted in a decision tree for calculating if the vertical distribution is vertically uniform, Gaussian, exponential, or hyperbolic. Remote sensing presents different problematic aspects that make it not suitable for monitoring irrigation wells. The main one is that the resolution of the images is too small to allow the monitoring of irrigation infrastructures.

The use of sensors for the monitorization of water quality has several advantages compared with remote sensing. They are not affected by clouds which impedes the direct observation of the earth surface. Moreover, the sensors have a small since and can be deployed in different parts of the irrigation infrastructure regardless of the dimensions of the monitored area. Finally, the sensors can be located at different depths to monitor the water column itself. The use of a turbidity sensor to control the transparency of the water is one option to evaluate eutrophication. We find a few examples of sensors used to monitor the presence of sediment or algae. Parra et al. [18] proposed a system based on 4 LEDs (red, yellow, green, and infrared) and light receptors. Their prototype was tested with two species of phytoplankton (green and brown) and sediment. They use an algorithm that determines the turbidity with the infrared led. Afterwards, the red led is used for determining the type of algae or sediment. The prototype proposed in [18] is similar to our prototype. Nevertheless, the proposed prototype uses more LEDs to improve the sensor’s performance. Specifically, the proposed sensor is based on the light absorption of six LEDs; five of them belong to the visible spectra and the last one to infrared wavelength. The reason for choosing this number of LEDs is that they allow incrementing the sensed data compared with the previous prototype while maintaining a reduced number of node inputs and outputs. Therefore, we will measure mixed samples to distinguish the turbidity sources in complex scenarios. Moreover, we present the developed communication system of our sensor.

## 3. System Description

In this section, the proposed sensor node, as well as the communication protocol, is presented. Regarding the prototype, we describe the employed materials for its creation and its features. Secondly, we compare and select the best communication technology for our scenario. Finally, we identify the most suitable node to accomplish the requirements of this prototype in terms of input/output pins and the technology selected.

### 3.1. Characterization of the Prototype

The prototype used in this experiment is crafted in PVC and glass. The PVC has been selected because of its robustness and watertight properties, making it suitable for high durability. The glass is used to contain the water to be measured; it is selected because of its transparent characteristics that will allow the path of light through the sample. The developed prototype is characterized by its modularity, which ensures that damaged elements can be easily replaced. The casing is performed with a 20 cm T-shaped PVC. This external module has an internal and outer diameter of 4 cm and 5.6 cm, respectively.

The internal section, where the sensing elements are placed, is made of PVC, with a length of 18 cm and a thickness of 0.3 cm. The inner diameter of this tube is 2.6 cm, and the external diameter is 3.2 cm. A total of 12 holes, to place the sensing elements, have been made in the inner tube, divided into two groups of six holes placed at 180° of each other. The space between holes in each group is 1 cm. On the one hand, as a light emitter, six LEDs (blue, green, yellow, orange, red, and infrared) are inserted in one of the groups of holes. On the other hand, as light receptors, five LDRs are used for the visible light, and a photodiode is selected for the NIR light. Although six lights are included in the prototype, we focus on the visible lights in this paper. In our prototype, we select the use of LDR for visible light. The LDRs do not present response times as fast as photodiodes. In real conditions, this is an advantage since sudden changes will less influence the sensor’s response in incident light. The possible sudden changes might include the pass of fauna, rocks or flocs across the light pass. Furthermore, eutrophication processes are slow processes concerning the response time of an LDR. Therefore, the response time of our light detectors is appropriate to evaluate the algal growth. Finally, the electronic circuit of the LDR is more straightforward than the photodiode circuit, facilitating maintenance, repair, and construction tasks. The infrared light has been widely studied in other prototypes; it will be used only to estimate the concentration of solids [18]. The cover of the upper part of the sensor has been modified by creating a perforation to include the glass vial. This vial has an internal diameter of 0.9 cm and a length of 1.5 cm. Waterproof silicone was used to seal the space between PVC and glass vial. The vial is aligned between the light emitters and receptors, allowing to measure the absorption of light without direct contact between sensing elements and water.

**Figure 1:**
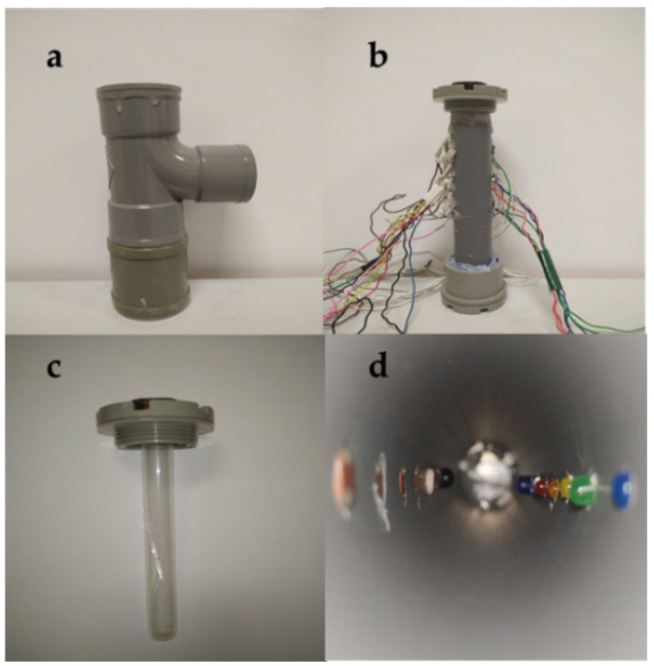
Representation of the used prototype: (**a**) Casing; (**b**) Prototype; (**c**) Sample vial; and (**d**) Position of LEDs and LDRs.

Figure 1 presents the different sections of the developed prototype. Figure 1a represents the encapsulation of the prototype, Figure 1b the core of the sensor, and Figure 1c the vial where the sample is deposited. Finally, Figure 1d displays the arrangement of the LEDs and LDRs inside the prototype. Table 1 summarizes the dimensions of the prototype, and Table 2 outlines the characteristics of selected LEDs.

### 3.2. Selection and Definition of Communication Technology

One of the most critical capabilities that must be considered when selecting and implementing sensor nodes is the communication system or protocol that will be used to transmit the monitored information through the nodes. The nodes will be installed in irrigation ponds, and, as these ponds are generally located at a great distance from the points from which it is possible to connect to Internet Service Providers (IPSs). Therefore, this connection becomes very complicated, and to solve it, it will be necessary to use communication through wireless technologies.

Among the wireless technologies most used in the IoT and Agriculture environment [25] we can highlight the following ones (i) Wi-Fi (IEEE 802.11) [26], (ii) Radio-frequency identification (RFID) [27], (iii) Bluetooth Low Energy (BLE) (IEEE 802.15) [28], (iv) ZigBee (IEEE 802.1.4) [29], (v) Sigfox [30], (vi) LoRa [31], and (vii) mobile technologies, such as WiMAX (IEEE 802.16) [32], LTE Cat-M1 [33] or NB-IoT (5G) [34]. Studying the previously listed technologies, we can affirm that there is no better or worse technology than another. Still, its operation will depend on the environment in which it will be applied. Therefore, when implementing a technology, multiple factors must be considered, such as the distance between nodes, the existence of obstacles in the environment, the amount of data to be transmitted, the energy autonomy of the system, the cost of performing the deployment, etc.

In our case, we can discard technologies with a more limited scope, such as RFID, BLE, or Zigbee. Moreover, we can discard those with a higher maintenance cost, since, although can operate at a greater distance, which has an additional cost of connection to an ISP, such as WiMAX, LTE Cat-M1, or NB-IoT. For all the reasons previously described, the most appropriate technologies to carry out our implementation are Wi-Fi and LoRa. The best option for this case is the combination of both technologies creating a Hybrid solution using Wi-Fi and LoRa technologies.

**Figure 2:**
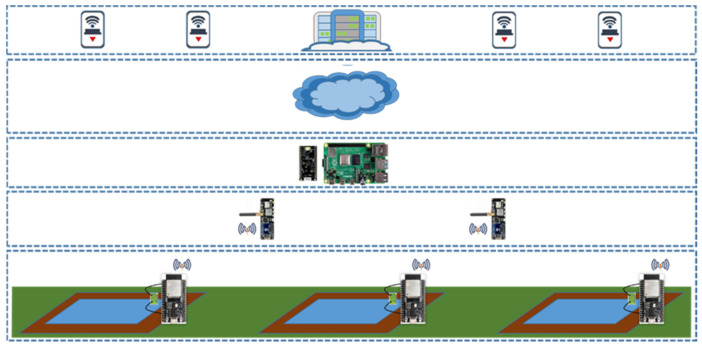
Architecture employing Wi-Fi and LoRa Technologies.

For some assumptions, the ideal solution may be implementing both Wi-Fi and LoRa technologies in different layers of the architecture. The sensors will be connected to ESP32 Wi-Fi nodes, using Wi-Fi technology, from these nodes, the data will be sent to TTGO T-Beam ESP32 Wi-Fi GPS NEO-6M LoRa 868 MHz nodes [35], in this way, the nodes can receive the data that have been obtained during motorization and, in turn, can be transmitted using LoRa technology to LoRa gateways. Once the gateway is reached, the same action is taken as in model 2. The LoRa gateway will be connected to a Raspberry Pi 4. From the Raspberry Pi 4, the LoRaServer network and application servers will startup, which will be from where they will respond to the demands of users who will be using a real application to monitor the system or the cloud. Figure 2 presents the layers of the communications architecture where the sensors will be located. At the lower level, the technology used is Wi-Fi. The next level receives the information from the lower level by Wi-Fi and transmits it to the upper level by LoRa. At the next level, we find the LoRa gateway together with the LoRaServer network and application servers. Finally, we see the same devices we present in model 2 described above in the upper levels.

### 3.3. Description of the Selected Node

**Figure 3:**
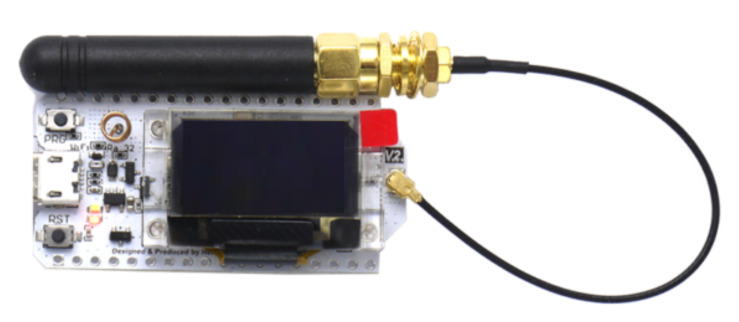
Node Heltec Wi-Fi LoRa 32.

In this subsection, we describe the nodes that we will use in the irrigation reservoirs. The nodes proposed for our implementation are from the manufacturer Heltec Hel-tec Automation (TM). They are part of the Heltec LoRa series and are the Wi-Fi LoRa 32 model [36]. Figure 3 shows an image of the sensor node.

The node has been selected mainly for characteristics such as its transmission capacities, connection possibilities and power characteristics. Of its main features, we can highlight the following ones:

- Microprocessor: ESP32 (240 MHz Tensilica LX6 dual-core + 1 ULP, 600 DMIPS);
- Wireless Communication: Wi-Fi, LoRa, Bluetooth. (802.11 b/g/n; Bluetooth V4.2 BR/EDR and BLE; node-to-node communication or LoRaWAN);
- LoRa bands: EU_433, CN_470_510, EU_863_870, us_902_928;
- Hardware Resource: UART × 3; SPI × 2; I2C × 2; I2S × 1; 12-bits ADC input × 18; 8-bits DAC output × 2; GPIO × 22, GPI × 6;
- Battery: 3.7V Lithium (SH 1.25 × 2 socket);
- Energy: (Power supply (5 V, 3.7 V, 3.3 V); Output (5 V, 3.3 V).

This node powers the LEDs and measures the change of resistance in the LDRs. In addition, the select node has a LoRa and Wi-Fi connection which allows the intercommunication of the nodes with a central server.

## 4. Test Bench

This section describes the equipment used to perform the tests and the followed methodology selected for the entire data collection, calibration, and verification process.

### 4.1. Instrumentation

First, we describe the equipment used to power the systems and measure the sensing elements’ output signal, the light detectors. To power the LEDs, a FAC 662-B generator [37] has been used. LEDs are fed at 5 V with continuous current, resistors of 470 Ω have been used. Regarding the light receptors, in this paper, we focused on the use of visible light. Therefore, to measure the response of the LDRs, a multimeter TENMA 72-2600 [38] have been used.

To prepare the sample, a precision balance ENTRIS II model BCE223i-1x [39] was used to measure the weight of sediments and algae. To create the samples, tap water was used and a graduated cylinder with a volume of 100 mL and a precision of 1 mL.

### 4.2. Samples Preparation

In this subsection, the process followed for the calibration of the sensor using different samples is detailed. A total of seven samples were prepared, two pure samples (1 of sediment and one of *Chlorella vulgaris* (as algae)) and five mixed samples with different percentages of each turbidity source. The combinations used are: 80% sediment/20% algae; 60% sediment/40% algae; 40% sediment/60% algae; 20% sediment/80% algae. Drinking water from the tap was used to perform the samples. For these samples, sediment or algae are weighted to prepare the solution with a concentration of 7142 mg/L. The volume of the solutions is 100 mL. A serial dilution process is applied to generate the eight dilutions the each one of the solutions. The concentrations of the solutions are: 0 mg/L, 15 mg/L, 50 mg/L, 200 mg/L, 800 mg/L, 1500 mg/L, and 4000 mg/L.

### 4.3. Measuring Methodology

Once we have obtained the different samples and their dilutions, 25 mL of each is used to take measurements with the prototype; 25 mL is the volume of the sensor vial. The aliquots are transferred to the vial, and the measurement process starts. The LEDs are powered sequentially (blue-green-yellow-orange-red), and the resistance of the associate LDR is measured to gather the data. The value of the LDR requires a short time to stabilize its measure. The data were collected once the value was stable. After each measurement, the LED is turned off, and the subsequent LEDs is powered. Using this methodology, we obtain the data as resistance values (kΩ). A voltage divider has been simulated to attain the results in units of voltage (V). The exact values of the voltage divider are presented in the results.

## 5. Results

In this section, the obtained results are presented. First, we compare the resistance values gathered in the different LDRs. Following, we transformed the resistance of the LDRs to voltage and evaluated the sensor’s calibration with varying models of regression. Finally, we test the use of an artificial neural network to enhance the accuracy of the classification of samples.

### 5.1. Resistance of LDRs with the Use of a Combination of Turbidity Sources and Lights

This section analyses the resistance values obtained for the different concentrations tested of sediment, *Chlorella vulgaris*, and their mixture with other lights in the LDRs used. First, we elaborated a multifactorial analysis of variance (ANOVA) to determine if there are significant differences between the type of turbidity and the concentration of solids. This information will indicate whether using the prototype to differentiate turbidity sources at different concentrations is possible. In Table 3, the results of the multifactorial ANOVA are shown. We observe that the *p*-values are below 0.05 in all the cases. Thus, we can determine that there are significant differences in the resistance values of the LDRs between the different types of turbidity sources and the tested concentrations. This indicates that it is feasible to use the gathered data to distinguish the turbidity concentration and its origin.

Next, we evaluated these differences. Therefore, we assess the resistance values obtained for the different turbidity sources and concentrations. The figures below represent the resistance values of the LDRs for the different light sources. Although the *X*-axis represents the tested lights, the levels of concentration are represented in *Y*-axis logarithmically. In general terms, we can observe an exponential growth of the resistances with the increase in turbidity, except with the red and orange lights. In these cases, the resistances values decrease between 0 to 15 mg/L in all the samples tested and increase again until 4000 mg/L. This effect might be produced by the different reflections and refractions of light in the glass compared with other LEDs due to slight differences during the assembling. In future work, we will change the design of the prototype to avoid that light can be travel outside the glass. For this, we will add two sheets of non-translucent material between the glass and the PVC walls. Moreover, the vial containing water will have a squared section instead of a round section.

**Figure 4:**
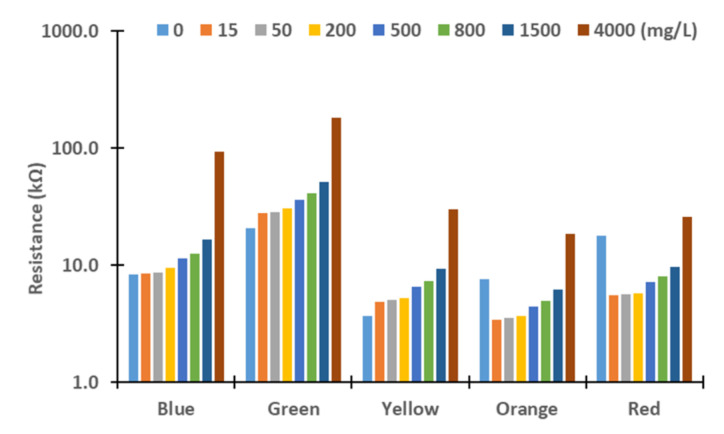
Measured resistance values for sediment.

**Figure 5:**
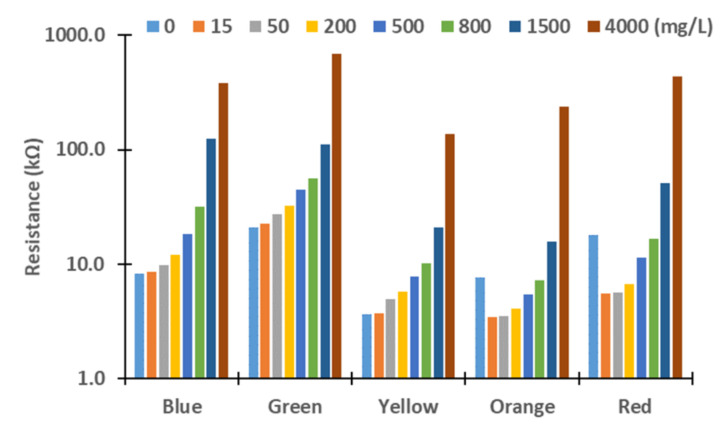
Measured resistance values for algae.

In Figure 4 and Figure 5, measured resistance values of LDRs are represented for the different tested concentrations of sediments, see Figure 4; and *Chlorella vulgaris*, see Figure 5. Except for low concentrations, 15 and 50 mg/L, gathered resistance values are higher when *Chlorella vulgaris* is used as a turbidity source for all light sources. The observed differences are greater for the highest concentrations. For the green light, and especially for the concentration of 15 mg/L, there is a big difference in the resistance values, 22.5 kΩ in algae turbidity and 27.93 kΩ in sediment. Regarding the concentration of 50 mg/L, the values of the resistance values for algae and sediments are similar, with values of 27.5 to 28.3 kΩ, respectively. The same phenomenon occurs when the yellow, orange, and red lights are used. Resistance of LDRs presents a high difference between the algae and the sediments in the concentration of 15 mg/L. Nonetheless, for the concentration of 50 mg/L, the resistance values are similar regardless of the turbidity source. This might occur due to the liberation of pigments of the algae into the water, causing an increase in light absorption at specific wavelengths.

**Figure 6:**
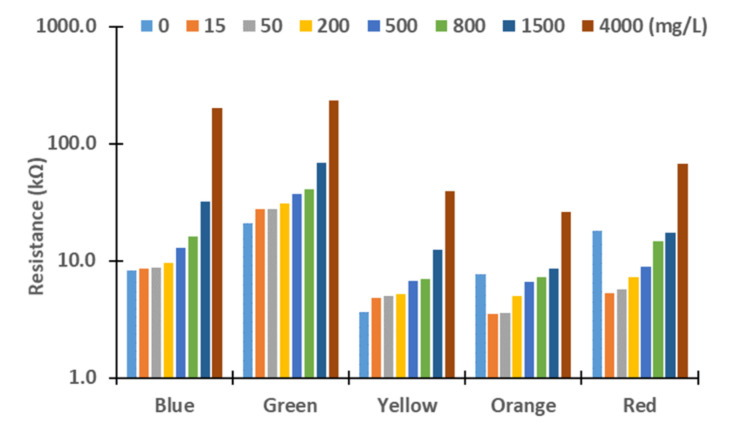
Measured resistance values for mixture 80% sediment and 20% algae.

**Figure 7:**
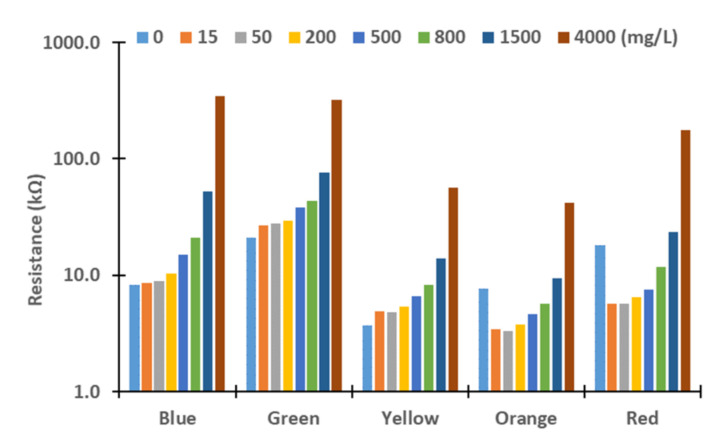
Measured resistance values for mixture 60% sediment and 40% algae.

**Figure 8:**
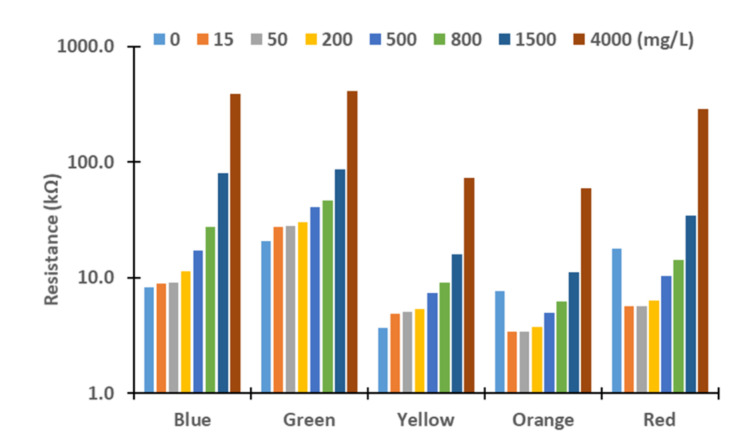
Measured resistance values for mixture 40% sediment and 60% algae.

**Figure 9:**
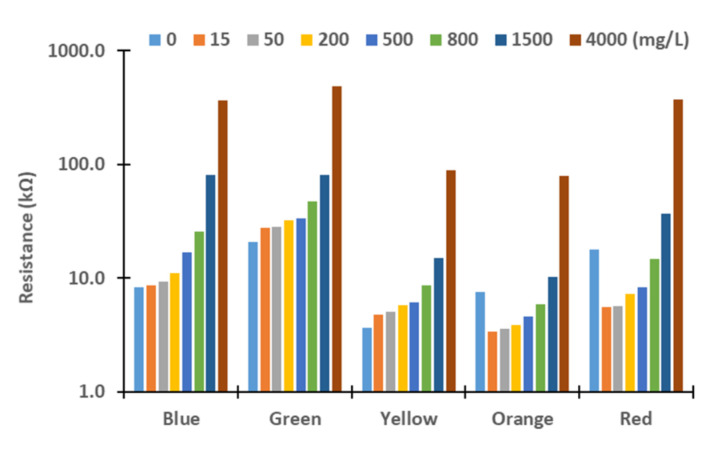
Measured resistance values for mixture 20% sediment and 80% algae.

In Figure 6, Figure 7, Figure 8 and Figure 9, resistance values of the different combinations of turbidity sources for the different lights are represented. It is to be expected that the resistance values of mixed samples are between the resistances values of sediment and algae. In general terms, resistance values are between the resistance of pure sediment and algae. When resistance values are out of the expected interval, the gathered values are similar to the values of sediment or algae. As occurred in results of pure samples, the increase in solids concentrations in mixed samples led to an upsurge of the LRDs resistance values in all cases (except the rise from 0 to 15 mg/L in red and orange LEDs).

### 5.2. Calibration of the Sensor for Different Sources

In this subsection, we describe the calibration models for the different lights and turbidity sources. First of all, resistances are converted into voltages, which is the variables measured by the node. Then, we use Statgraphics Centurion XVIII [40] to obtain the mathematical models that correlate the solids concentration with the input voltage at the node, which is the output voltage of the voltage divider.

To simulate the operation of the node, we use a voltage divider, see Equation (1), to determine the change of voltage in the node input due to the differences of resistance of the LDRs. Where input voltage (IV) is the voltage provided by the node, the output voltage (V_out_) is the voltage after the voltage divider (the input voltage for the node), the circuit resistance (CR) is the value of second resistance, the value we will adjust, and the LDR resistance (R_LDR_) is the resistance of each one of the LDRs of the prototype. As IV, we use 3.3 V since it is the typical voltage of pins in different microcontrollers as Arduino or Raspberry.

(1) $${V\;}_{\mathrm{out}}\left(V\right)=\frac{\mathrm{IV}\;\left(V\right){*\;R}_{\mathrm{LDR}}\left(\Omega \right)}{\mathrm{CR}\;\left(\Omega \right)+{\;R}_{\mathrm{LDR}}\left(\Omega \right)}$$

To improve the sensibility of our sensor, we select a CR that maximizes the voltage difference between 15 mg/L and 500 mg/L and for a particular turbidity source. We delete the concentration equal to 0 mg/L of the calibration. Thus, we choose 15 mg/L as the minimum expected concentration. We select 500 mg/L because we do not expect the concentration of sediments to exceed 500 mg/L in normal conditions. We need to choose one of the different turbidity sources to maximize the differences. Sediment was selected as a turbidity source since it has the smallest difference between the minimum and maximum resistance values. Considering that resistances calculated with Equation 1 are not standard resistance values and those resistances are hard to obtain, we use the most similar standard resistance. In Table 4, there are the values of resistance obtained by Equation (1) and the standard values used.

Once we have established the fixed resistance values in the voltage divider, we can calculate the node’s voltage values. It is important to note that the node cannot measure all values of the analogic signals. The analogic pins convert the signal from analogue to digital. For this function, the node has a limited number of values (10 bits). Therefore, we must convert the analogue values obtained with Equation (1) into digital values with 10 bits of resolution. First, we determine the minimum input voltage in the node when the different light sources are used. We established that the maximum voltage is 3.3 V, which is the maximum value that Vout can reach. After that, we calculate the difference between the minimum and maximum voltage for each light. This value is divided into 1024 (10 bits), and we obtain the accuracy of the reading to calculate the new voltage values (Equation (2)). In Equation (2), Voutc is the new corrected voltage. Vout is the value obtained in Equation (1), and precision is the precision of the input for the light source used. In Table 5, we show the calculated precision of the analogy.


(2) $$V_{\mathrm{outc}\;}\left(V\right)=\mathrm{Integer}\left(\frac{V_{\mathrm{out}}\left(V\right)}{\mathrm{Precision}\;\left(V\right)}\right)*\mathrm{Precision}\;\left(V\right)$$

The following step is to have a mathematical model that correlates the concentration of both two types of solids (sediment and algae) in the water with the gathered Vout in the node when one or more lights are used. We use Statgraphics Centurion XVIII [40] to generate the regression models. We search for the best model with the higher R2 coefficient that relates them. Our objective with the models was to determine the concentration of one of the turbidity sources, alga in this case, and infer the turbidity of the second source based on the total concentration of solids in water. This is estimated using the infrared LED as it was described in [18].

**Figure 10:**
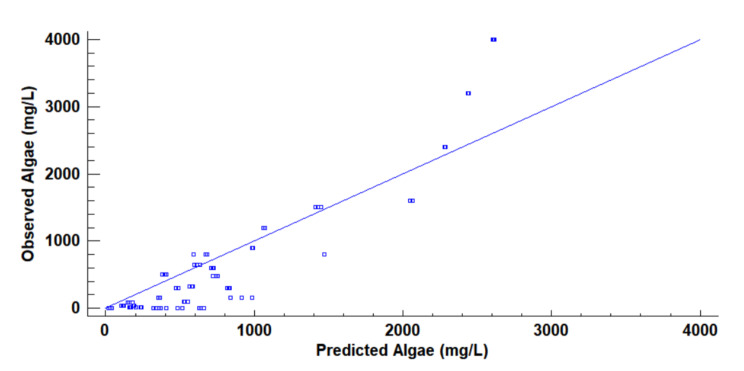
Observed versus predicted values of algae concentration with Equation (2).

With Statgraphics Centurion XVIII [40], we performed a multiple regression analysis to determine the model that related the Vout obtained with the different light sources with the turbidity source or sources. In the initial model, the voltage obtained with the orange light is not significant, with a *p*-value of 0.1818. We delete this parameter and the new model represented in Equation (3) with an R^2^ of 0.7721. In Equation (3), V_outB_, V_outG_, V_outY_, and V_outR_ represent the output voltage of blue, green, yellow, and red, respectively. The model of Equation (3) is modelled using all the gathered data (all turbidity sources and all tested concentrations). Figure 10 represents the predicted versus the observed values of the concentration of algae using Equation (3). We can observe that there are many differences between the predicted and the observed values.

(3) $$Algae\;\left(mg/L\right)=-1547.2-2654*V_{outB}\left(V\right)-7293.1*V_{outG}\left(V\right)+8523*V_{outY}\left(V\right)+2688*V_{outR}\left(V\right)$$

**Figure 11:**
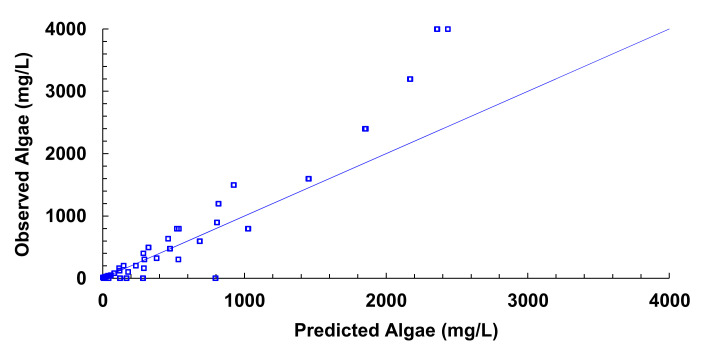
Observed vs. predicted values of algae concentration with Eureqa.

Another option is using Eureqa software [41] to obtain a mathematical model that correlated the gathered data. Eureqa is software that searches a model that adapts the observed values with a mathematical model. As previously, the orange light values are discarded. Thus, we do not include this variable in the Eureqa model. We select a model with low complexity (16 of Eureqa complexity value) and high R^2^ (R^2^ of 0.9542). The model used is represented in Equation (4). We observe that the parameter of voltage obtained with yellow light is not included in this model. The predicted versus observed values of algae concentration are represented in Figure 11. As in the previous case, the difference between predicted and observed is high.

The problem with this type of regression is caused because of the wide range of concentrations tested and the non-linear response. For this reason, we discard the use of multiple regression with all the concentrations. Therefore, it is necessary to reduce the variability of data to obtain better adjustments.

(4) $$Alga\;\left(\frac{mg}{L}\right)=498.6*V_{outB}\left(V\right)+\frac{-81.5}{V_{outB}\left(V\right)-3.3}-802.3-V_{outG}{\left(V\right)}^{2}$$

As the previous solution does not present a good performance, we decide to use different models according to the concentration of solids in the water. First, we determine the concentration of solids with an infrared LED and photoreceptor, as [18]. According to the concentration of solids, we will select a particular mathematical model. In the case of intermediate concentrations between the studied concentrations, an interpolation is performed.

**Figure 12:**
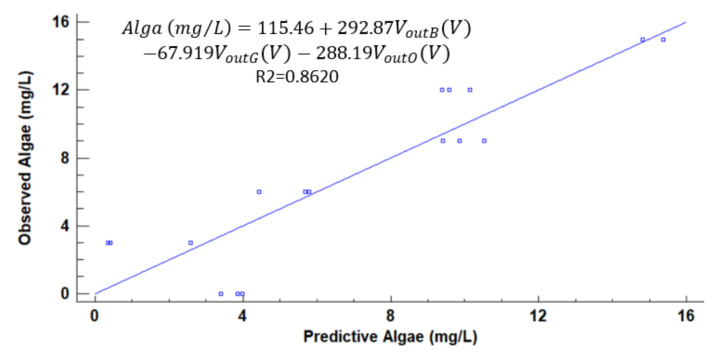
Observed vs. predicted values of algae concentration with a solid concentration of 15 mg/L.

**Figure 13:**
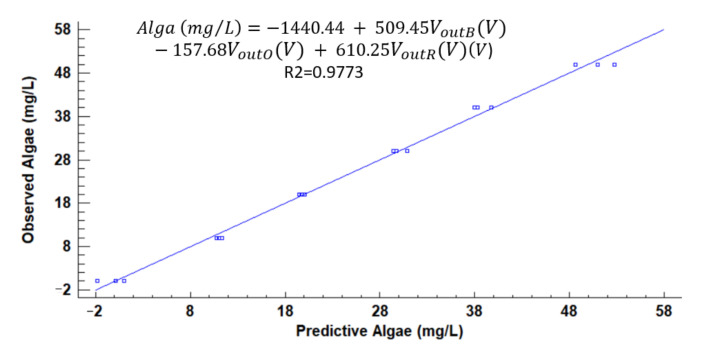
Observed vs. predicted values of algae concentration with a solid concentration of 50 mg/L.

**Figure 14:**
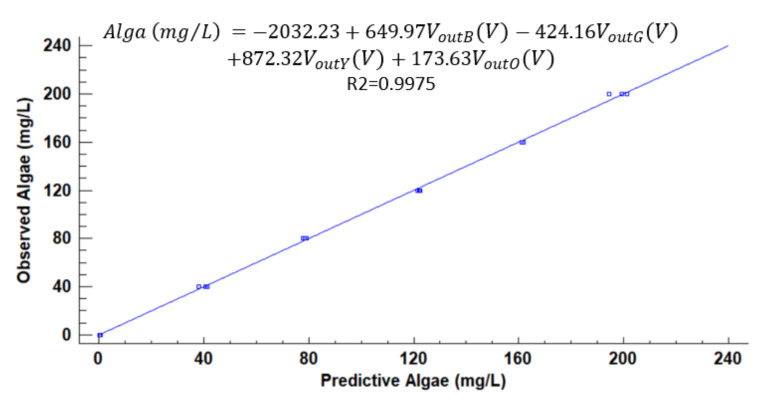
Observed vs. predicted values of algae concentration with a solid concentration of 200 mg/L.

**Figure 15:**
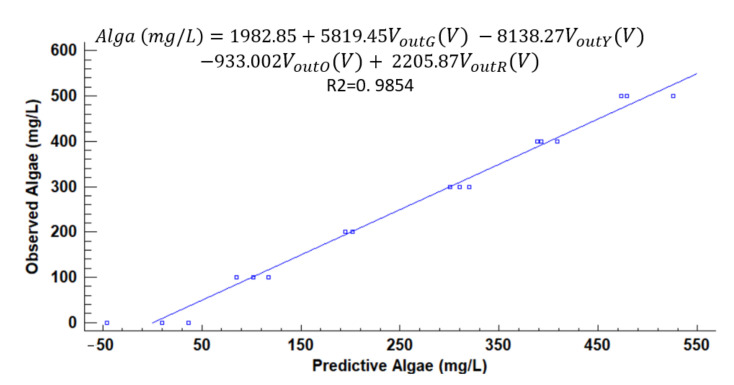
Observed vs. predicted values of algae concentration with a solid concentration of 500 mg/L.

**Figure 16:**
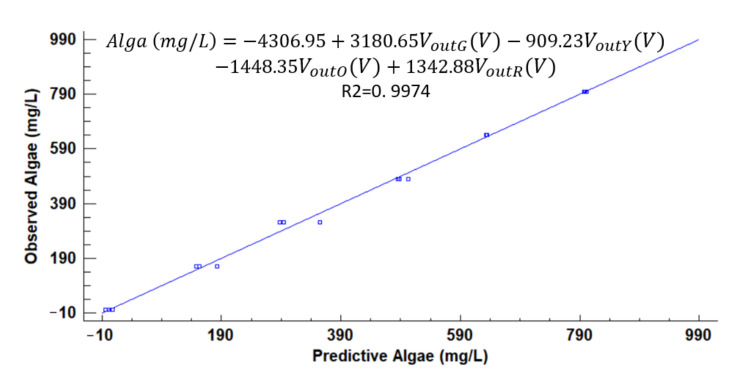
Observed vs. predicted values of algae concentration with a solid concentration of 800 mg/L.

**Figure 17:**
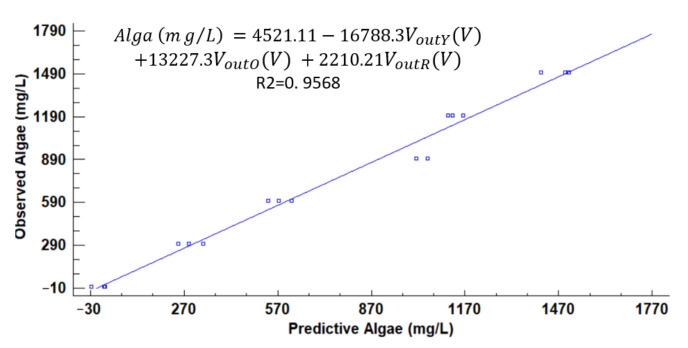
Observed vs. predicted values of algae concentration with a solid concentration of 1500 mg/L.

**Figure 18:**
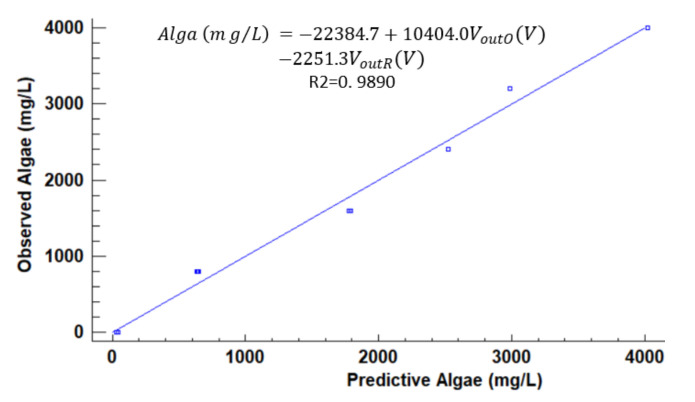
Observed vs. predicted values of algae concentration with a solid concentration of 4000 mg/L.

Figure 12, Figure 13, Figure 14, Figure 15, Figure 16, Figure 17 and Figure 18 represent the predictive versus observed algae concentration by each solid concentration. In addition, we can observe the mathematical model and R^2^. The lowest value of R^2^ is presented in the equation of 15 mg/L with a value of 0.8620, see Figure 12. In this concentration, predicted values differ from observed more than in other concentrations. The rest of the models used present good values of R^2^. The model calculated for 15 mg/L of solids present and a relative average error of 40%. The lower errors are in the concentration of 800, 500, and 200 mg/L with relative average errors of 4.9%, 5.2%, and 6.2%, respectively. The models for the concentration of 50 and 1500 mg/L are characterized by 12.1% and 10.0% of relative average error. Finally, the relative errors in the mix of sediment or algae are maximum in the concentration of 80% sediment and 20% algae, with a relative error of 32.1%. The rest of the samples with mixed turbidity sources have relative errors between 4.3% to 11.1%.

**Figure 19:**
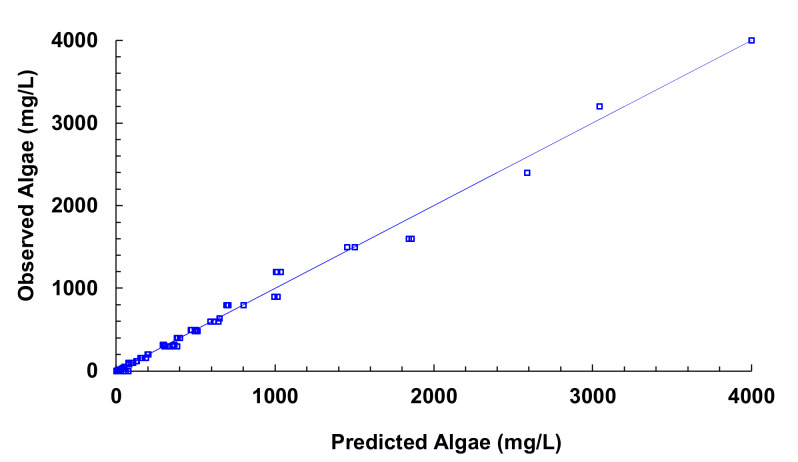
Observed vs. predicted values of algae concentration with different models.

Figure 19 illustrates the observed versus predicted values of voltage for all joined models. We can observe that the combined model presents good predictability of the algae values. The average error between the predictive algae concentration and observed is 36.52 mg/L and a relative error of 12.56%.

If we observe the different figures of the models presented, in some cases, we find that the concentration of algae is negative or higher than solid concentration. As this cannot happen in natural conditions, we apply two modifications to the results. On the one hand, if the result is a negative concentration, the result will be changed to 0 mg/L of Algae. On the other hand, if the result is an algae concentration higher than solid concentration, the result will be adjusted to solid concentration (100% algae). With these simple modifications, we reduce the absolute error to 31.95 mg/L and the relative error to 11.0%.

**Figure 20:**
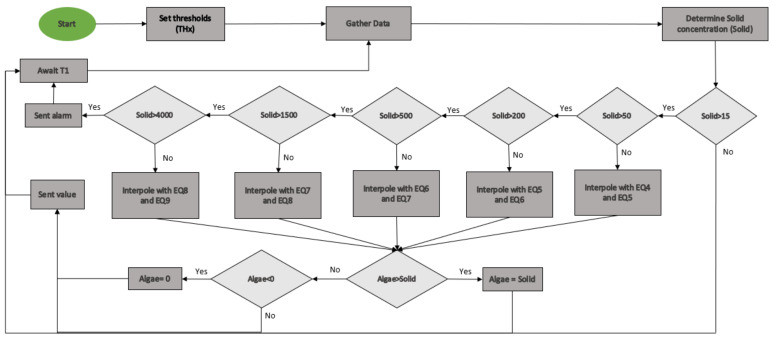
Prototype algorithm.

Finally, Figure 20 present the algorithm of the prototype. When the prototype starts the measure, we set the different thresholds. Then, we start the function loop. First, the data of the different photoreceptors is obtained. With infrared light, we determine the concentration of solids. If the solid concentration is lower than 15 mg/L, the prototype will be on standby for a time until the subsequent measurement. If the solid concentration is higher than 15 mg/L and lower than 4000 mg/L, the prototype will use the closest equations for the solids concentration and perform interpolation to obtain the algae concentration. Once the concentration of algae is obtained, the corrections are applied to verify that the concentration is greater than 0 and lower than or equal to the concentration of solids. Finally, this information is sent, and the prototype will go into rest until the subsequent measurement. If the concentration is higher than 4000 mg/L, the prototype sends an alarm. We assume that this concentration is too high for irrigation water. After sending the warning, as in the previous cases, the sensor will go into standby status.

With the multiple regression models adjusted for every tested concentration, it was possible to reduce the error and reach a reasonable adjustment that allows an optimal operation of the sensor. The computational requirements of including these models do not differ too much from the requirement for a single multiple regression model. The errors obtained in this calibration and the R2 are similar to those obtained in other papers describing low-cost sensors. For example, in [15], another optical-based prototype for water quality monitoring, relative errors between 4.9% and 19.6% are related, and R2 values are between 0.99 and 0.97. In this paper, unless R2 between this range characterizes the model for 15 mg/L, the rest of the models and the average relative error is also between the range of [15].

### 5.3. Artificial Neural Network

In the previous section, we study the use of different models to determine the concentration of algae. Now, we analyze the use of an artificial neural network to determine the percentage of algae in the different mixtures as an alternative to previous models. The aim of exploring this option is that some nodes might be endowed with the capability to use these systems in the short term to maintain low-cost devices and low-energy consumption. Therefore, we wish to compare the performance of existing solutions which can be included nowadays in the node with the nearby possibilities.

**Figure 21:**
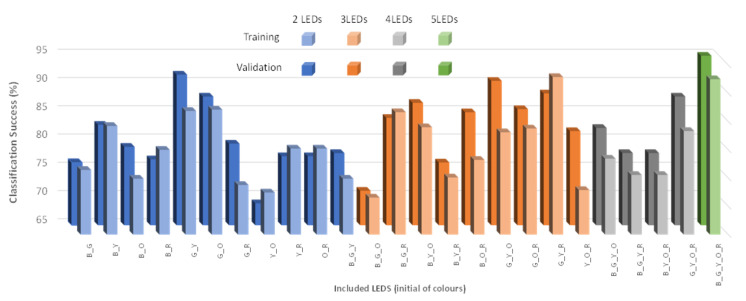
Training and validation of neural network.

To develop the analysis of a neural network, we use Statgraphics Centurion XVIII [40] software. For the training and validations phases, 90 and 36 random values are used. In order to include the possible effect of this random selection on the success, the analyses were performed five times. Figure 21 represents the values of training and validation success for different combinations of lights. We test the use of 2, 3, 4, and 5 lights. The best percentage of success is 93% with the use of three LEDs (green, yellow, and red); another high rate of success is for the use of the 5 LEDs with a value of 92%. In general terms, there does not seem to be a relationship between the use of a specific light source and a high success rate in the neural network. Regarding validation, the highest value is 98% with the use of 5 LEDs.

We have selected 5 LEDs for our neural network since it is the one with the highest percentage of success. Table 6 and Table 7 summarize the success for training and validation, respectively. In the case of training, the values of success are high. The group with less percentage of success is 60% of algae. In this group, two points are missclassified as 0% of algae. The same occurs in the group of 40% algae, where two other values have been classified in the group without algae. Regarding the validation, we observed 100% effectiveness when classifying the values.

The use of an artificial neural network implies a great demand for calculation. Although there are different proposals about implementing artificial neural networks in microcontrollers, we consider that the best option to monitor the presence of algae or sediments and their mixtures is the proposed algorithm. Even though the algorithm can be less efficient than an artificial neural network, it requires a lower calculation power. If better precision is required, the data can be sent to a computer to perform the neural network. This solution would improve the determination of the mixing percentage at the cost of higher energy consumption.

## 6. Conclusions

The measurement of water turbidity might not be enough in many cases since different turbidity sources might require other actions. For the same level of solids concentration, if turbidity is caused by sediment, it can indicate a high water runoff. In contrast, the algae might indicate a pollution event linked to eutrophication. Therefore, the characterization of turbidity is essential. There are no low-cost devices capable of measuring turbidity and distinguishing those suspended solids’ sedimentary and phytoplanktonic origin.

In this paper, we have developed and calibrated a low-cost sensor based on light absorption capable of quantifying the concentration of algae and sediment in mg/L independently. The prototype is based on the light absorption of 5 LEDs with different light colors. Two operational methodologies for the prototype operation are given and compared. On the one hand, the use of multiple regression models adjusted for different solids concentrations is proposed. The combined model (a combination of seven multiple regression models) is characterized by low absolute and relative error, similar to those of other low-cost sensors, which possibilities its use. On the other hand, for a more powerful node, using an artificial neural network ensures a drastic reduction in the errors with a correct classification of 100% of the cases in the validation phase. The communication technology for this prototype and a possible node for its control are proposed.

Future work will include other alga species, specifically brown algae, as in [18], to evaluate if models can be adapted to different types of algae. In addition, the combination of this sensor with other low-cost sensors for water quality monitoring will be performed to define a sensor node for developing a water quality observatory.

## Figures and Tables

**Table 1:** Dimensions of each section of the prototype.

	Encapsulate	Nucleus	Vial
External diameter (cm)	5.6	3.2	1.5
Internal diameter (cm)	4	2.6	0.9
Length (cm)	20	18	16
Thickness (mm)	80	30	5

**Table 2:** Characteristics of the LEDs of the prototype employed in these tests.

Characteristics	Blue	Green	Yellow	Orange	Red
Wave length (nm)	467–476	562–575	581–594	581–618	612–625
Diameter (mm)	3	3	3	3	3

**Table 3:** Multifactorial ANOVA for the LDR resistances in the different lights. * Significant difference.

	*p*-Value for the Evaluated Factors of Turbidity	
Light color	Turbidity source	Concentration of turbidity
Blue	0.0000 *	0.0000 *
Green	0.0004 *	0.0000 *
Yellow	0.0003 *	0.0000 *
Orange	0.0011 *	0.0000 *
Red	0.0006 *	0.0000 *

**Table 4:** Mathematical and standard resistance to fixed resistance in the voltage divider.

Light Source	Blue	Green	Yellow	Orange	Red
Mathematical resitance (kΩ)	9.7	27.6	4.9	3.9	6.3
Standard resistance (kΩ)	10.0	27.0	5.1	3.9	6.8

**Table 5:** The precision of the analogy entry.

	Blue	Green	Yellow	Orange	Red
Minimum voltage (V)	1.497	1.438	1.381	1.521	1.441
Maximum voltage (V)	3.30	3.30	3.30	3.30	3.30
Difference (V)	1.803	1.862	1.919	1.779	1.859
Precision (mV)	1.76	1.82	1.87	1.74	1.82

**Table 6:** Correctly classified cases in training dataset.

Actual Group	Predicted Group						Correctly Classified
	0	20	40	60	80	100	
0	13	1	0	0	0	0	92.86%
20	1	16	0	0	0	0	94.12%
40	2	0	13	0	0	0	86.67%
60	2	0	0	12	0	0	85.71%
80	0	0	1	0	14	0	93.33%
100	0	0	0	0	0	15	100%

**Table 7:** Correctly classified cases in the validation dataset.

Actual Group	Predicted Group						Correctly Classified
	0	20	40	60	80	100	
0	7	0	0	0	0	0	100%
20	0	4	0	0	0	0	100%
40	0	0	6	0	0	0	100%
60	0	0	0	7	0	0	100%
80	0	0	0	0	6	0	100%
100	0	0	0	0	0	6	100%

## Data Availability

The data presented in this study are available on request from the corresponding author. The data are not publicly available due to privacy constraints.
